# Octave bandwidth photonic fishnet-achromatic-metalens

**DOI:** 10.1038/s41467-020-17015-9

**Published:** 2020-06-25

**Authors:** Abdoulaye Ndao, Liyi Hsu, Jeongho Ha, Jun-Hee Park, Connie Chang-Hasnain, Boubacar Kanté

**Affiliations:** 1 Department of Electrical Engineering and Computer Sciences, University of California, Berkeley, CA 94720 USA; 2 Department of Electrical and Computer Engineering, University of California San Diego, La Jolla, CA 92093-0407 USA; 3 Materials Sciences Division, Lawrence Berkeley National Laboratory, 1 Cyclotron Road, Berkeley, CA 94720 USA; 4 Department of Mechanical Engineering, University of California, Berkeley, CA 94720 USA

**Keywords:** Metamaterials, Nanophotonics and plasmonics, Sub-wavelength optics

## Abstract

Planar structured interfaces, also known as metasurfaces, are continuously attracting interest owing to their ability to manipulate fundamental attributes of light, including angular momentum, phase, or polarization. However, chromatic aberration, limiting broadband operation, has remained a challenge for metasurfaces-based optical components and imagers. The limitation stems from the intrinsic dispersion of existing materials and design principles. Here we report and experimentally demonstrate polarization-independent fishnet-achromatic-metalenses with measured average efficiencies over 70% in the continuous band from the visible (640 nm) to the infrared (1200 nm). Results of the scalable platform are enabling for applications requiring broad bandwidth and high efficiency including energy harvesting, virtual reality and information processing devices, or medical imaging.

## Abstract (web summary)

Here the authors demonstrate all-dielectric fishnet-achromatic-metalenses from the visible to the near-infrared region. This metalens performs efficiently independent of polarization over about an octave from 640 nm to 1200 nm.

## Introduction

In 1660, Newton published the discovery on the decomposition of white light by prisms and color theory. Since then, optical dispersion has continued to fascinate the scientific world. Dispersion is used in cosmology to measure the expansion of the universe via the propagation of gamma rays^1^. It is also used in many optical applications including mode-locked lasers and prism spectroscopy to cite a few, and, explains rainbows^2^. However, dispersion is a major challenge for imaging systems^3^. Chromatic aberration, originating from the variation of the refractive index of materials with frequency, limits the performance of broadband optical devices. The quintessential feature of chromatic aberration is the wavelength-dependent focal length leading to axial aberration that degrades the quality of images. To overcome this limitation, conventional optical-imaging systems often use an appropriate combination of lenses, such as the achromatic doublet or a combination of refractive and diffractive elements with opposite dispersion. Recently, interest has turned towards thinner, lighter devices known as metasurfaces^4–8^. They are subwavelength nanostructured interfaces, capable of controlling optical waves. A large variety of components has been reported, including lenses^9–16^, holograms, quarter-wave plates, half-wave plates, vortex plates, carpet cloaks, concentrators, polarizers, thin absorbers, or sensors^17–31^. Despite exciting findings, achieving simultaneously high efficiencies and large bandwidths has remained a challenge. Recent state of the art results in the visible reported a bandwidth from 470 to 670 nm with an efficiency of 20%^14^, while in ref. ^15^ the efficiency of 40% was obtained for the bandwidth from 400 to 660 nm.

Here, we experimentally report polarization-independent, fishnet-achromatic-metalenses (FAM) with measured average efficiencies over 70% in the continuous band from the visible (640 nm) to the infrared (1200 nm). The design approach is based on the simultaneous control of the slope and the phase-shift-intercept, two parameters that need to be continuously optimized in the lens for achromatic operation. The lens is fabricated in TiO_2_ and experimental Strehl ratios larger than 80% are measured in the entire octave bandwidth demonstrating diffraction-limited operation.

## Results

### Conditions for broadband achromatic operation

**Fig. 1:**
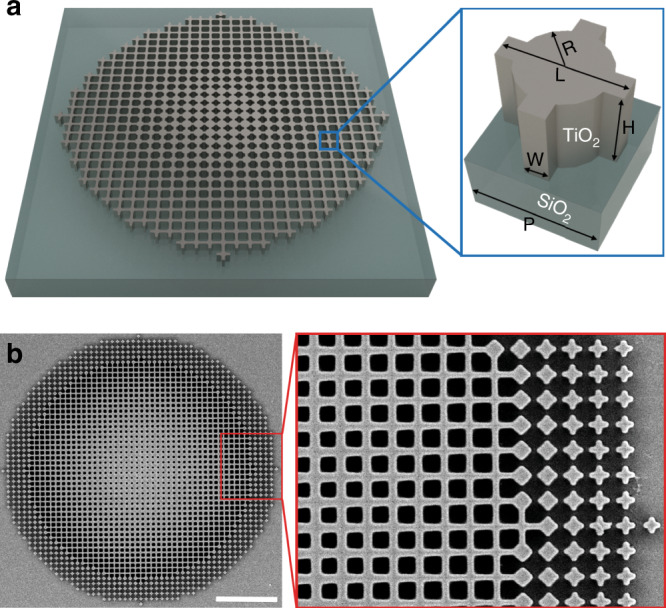
Schematic and scanning electron micrograph of a fishnet-achromatic-metalens (FAM). **a** Schematic of the proposed broadband metalens and its unit-cell with multi-degrees of freedom. The period of the unit-cell (P) is 370 nm, the height of the unit-cell (H) is 350 nm, and the metasurface is made of titanium dioxide (TiO_2_). The design parameters are the width of the cross (*W*), the length of the cross (*L*), and the radius (*R*). **b** Top view of an optical microscope image of a fabricated FAM and zoom-in showing the quality of the fabricated device. The scale bar represents 5 μm.

In order to focus light to a point for a normal incident plane wave, a flat lens needs to deflect light by a position (*r*) dependent angle (*θ*) given by the relation^5^:

1 $$\begin{array}{*{20}{l}} {{\mathrm{sin}}\left( \theta \right)} \hfill & = \hfill & {\frac{r}{{\sqrt {r^2 + F^2} }} = \frac{1}{{k_0}}\frac{{{\mathrm{{d}}}\phi \left( {r,f } \right)}}{{{\mathrm{{d}}}r}},{\mathrm{i.e.}},} \hfill \\ {\phi \left( {r,f } \right)} \hfill & = \hfill & {{\int} {\mathrm{{d}}} rk_0\frac{r}{{\sqrt {r^2 + F^2} }} = - \frac{{2\pi f}}{c}\left( {\sqrt {r^2 + F^2} - F} \right) + g,} \hfill \end{array}$$

where *ϕ* (*r*, *f*) is the phase profile required, *f* is the frequency, *F* is the focal length, *r* is the radial position, *c* is the speed of light, and *g* is a reference phase function independent of *r*. The reference phase can be an arbitrary function of frequency because only the spatial phase difference matters for the interference of waves at the same frequency after their interaction with the lens. We thus consider the phase shift, i.e., the phase difference between the local phase and the phase at the reference position taken at *r* = 0 (center of the lens). Hence, the phase-shift equation for a normal incident wave Δ*ϕ*(*r*, *f*) is

2 $$\begin{array}{*{20}{l}} {\Delta \phi \left( {r,f} \right)} \hfill & = \hfill & {\phi \left( {r,{\mathrm{f}}} \right) - \phi \left( {0,{\mathrm{f}}} \right) = - \frac{{2\pi }}{c}\left( {\sqrt {r^2 + F^2} - F} \right)}{\mathrm{f}} \hfill \\ \hfill & = \hfill & {m\left( r \right)f,m\left( r \right) = - \frac{{2\pi }}{c}\left( {\sqrt {r^2 + F^2} - F} \right),} \hfill \end{array}$$

where *m*(*r*) is the frequency slope of the phase-shift. Equation (2) reveals the requirements of a broadband achromatic metalens. First, the phase-shift for all positions is linear with respect to frequency. This condition can be locally satisfied using waveguide modes. Second, the slope of the phase-shift (dispersion) varies with position following Eq. (2) and the phase-shift Δ*ϕ*(*r*, *f*) is proportional to frequency, i.e., the phase-shift intercept with respect to frequency is zero. The metasurface is thus a waveguide array with, ideally, a local and simultaneous control of the slope and the intercept of the phase-shift. To satisfy the requirements of achromatic broadband metalenses, we propose to use the cross-circle waveguide shown in Fig. 1a as building block. The building block has four geometrical parameters that are the radius (*R*), the width (*W*), the length (*L*), and the period (*P*). Constraints impose for example *W* ≤ 2*R* and *L* ≤ *P*. Using geometric parameters, the slope can be controlled with a quasi-control of the phase-shift intercept consisting of minimizing it (ideally zero). Because each position has a unique (slope, phase-shift intercept) coordinate, dimensions can be chosen accordingly. One of the unique aspects of the device is that the design accounts for modified near-field interactions that usually hinder the performance of metalenses as explained in ref. ^23^. This is done via the iso-slopes and iso-phase-shift intercepts used in the construction of our metasurfaces. It is important to note that the four geometric parameters are not independent, as a change in any of them can affect the effective index of the waveguide they form. This signifies that it is challenging to have perfect achromaticity and efficiency as phase-shift intercepts and slopes cannot be fully independently controlled in a planar design. The limitation confirms that this is intrinsically an optimization problem^32–35^.

In metasurfaces, the spatial derivative of the slope controls the direction of incident rays to make them reach the focal point. It is thus important to have the correct slope to prevent chromatic effects and a decrease in efficiency. The intercept, however, controls the superposition of waves at the focal point, i.e., mostly affects the efficiency of the lens, not the position of the focal length. We can thus compromise on the intercept in the design of the lens. To quantify the impact of a non-zero phase-shift intercept on the efficiency of our metalens, Monte Carlo simulations are performed with 100 simulations for each element using a homemade finite difference time domain code. Each simulation was given a certain magnitude of the phase-shift intercept (error or deviation from the ideally zero phase-shift intercept) that was randomly distributed between unit-cells. The focusing efficiency was then compared to the ideal metalens implementing not only the correct slope but also the correct phase-shift intercept. Results, presented in Supplementary information, indicate that an error on the phase-shift intercept smaller than 30° decreases the efficiency of the metalens by <10% and does not affect the position of the focal point.

### Design of the FAMs

**Fig. 2:**
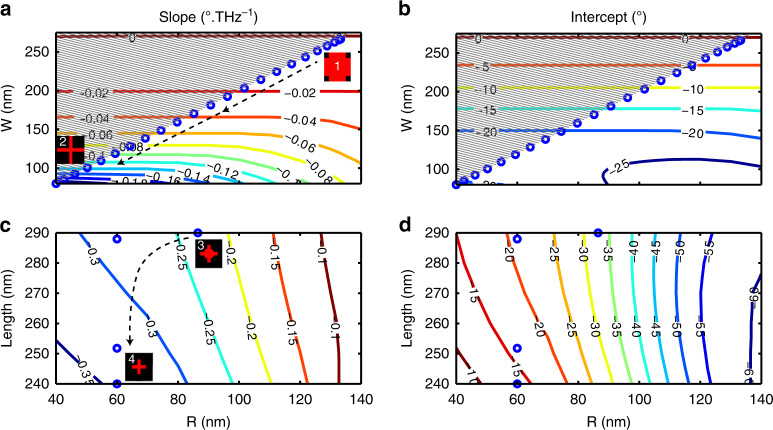
Design strategy of the FAM. To control the slope **a**, **c** and the phase-shift intercept **b**, **d**, at least two parameters need to be controlled. The considered two parameters are the width and the radius (*W*, *R*) (from step 1 to step 2 in **a**, **b**) and the length and the radius (*L*, *R*) (from step 3 to step 4 in  **c**, **d**). From step 1 to step 2, *W* varies from 80 to 270 nm, and, *R* varies from 60 to 135 nm while *L* is fixed to 370 nm. From step 3 to step 4, *L* varies from 240 to 290 nm while *W* is fixed to 80 nm to account for limitations in fabrication. Insets in **a** illustrate the evolution of the structure (top view) as one moves from the center (step 1) to the edge of the FAM (step 4), and, red/back colors represent TiO_2_/SiO_2_.

Figure 1a presents a sketch of the titanium dioxide (TiO_2_)-based metasurface and the geometry of the unit-cell with multiple degrees of freedom. It is a fishnet-like structure with a period *P* = 370 nm and a height *H* = 350 nm. The structure is fabricated by top-down nano-manufacturing methods and a scanning electron micrograph (SEM) of a fabricated metalens as shown in Fig. 1b. To design our metasurface, geometric parameters are controlled by pair, (*W*, *R*) in Fig. 2a, b and (*L*, *R*) in Fig. 2c, d. By considering fabrication limits, iso-slopes and iso-phase-shift intercept plots of realistic geometries are computed using full-wave numerical simulations (CST Microwave Studio) and the local phase method^23^, followed by least-square linear fitting. The phase shift of elements is calculated using a reference at the center of the lens with geometric parameters *W* = 270 nm, *R* = 135 nm, and *L* = *P* = 370 nm. For all other geometries, the parameters in Fig. 2 are calculated.

Figure 2a, c show that changes in *R*, *W*, and *L* enable slopes from zero to −0.35° THz^−1^ which in turn determines the maximum achievable size of the metalens for a given focal length. The figure also confirms that it is not possible to fully independently control the slope and the phase shift-intercept. However, accepting an error on the phase shift intercept enables designs sweeping all slope values. Figure 2a enables slopes from zero to −0.2° THz^−1^ while keeping a phase-shift intercept error below 30° (Fig. 2b). For the 20 μm × 20 μm metalens, we have chosen points indicated in blue (along the black arrow) to minimize discretization errors and points in the gray area are not geometrically allowed as *W* ≥ 2*R*. For absolute value of slopes larger than 0.2° THz^−1^, we used parameters in Fig. 2c and the second trajectory (blue points along the black arrow) also keeps the phase-shift intercept error below 30° (Fig. 2d). The evolution of the geometry of the unit-cell from the center of the lens to its edge is further discussed in supplementary information (Supplementary Fig. 1).

### Fabrication of the FAMs

The designed structure is fabricated by top-down methods and the SEM of a metalens, presented in Fig. 1b, clearly shows the high quality of the implementation. The fabrication uses three major steps. The first step consists of patterning the polymethyl methacrylate (PMMA) resist using electron beam lithography (EBL) that is subsequently developed in solution to remove the exposed PMMA. The pattern is the inverse of our final metasurfaces. In the second step, the exposed sample is transferred to an atomic layer deposition (ALD). The ALD process deposits 350 nm of TiO_2_ so that all features are filled. The third step consists of removing the residual TiO_2_ film that coats the top surface of the resist using reactive-ion-etching. After removing PMMA, the TiO_2_ metasurfaces were obtained. It is worth noting that FAMs have mostly connected structures and are thus more stable mechanically than metasurfaces based on fully disconnected elements. Fabrication imperfections with a magnitude of ±5 nm decrease the efficiency by at most 8%, making the FAMs robust (Supplementary Fig. 10).

### Characterization of the FAMs

The fabricated metalenses were optically characterized using a custom setup consisting of two main systems dedicated to illumination and imaging (Supplementary Fig. 5). The illumination system comprises a supercontinuum laser and an acousto-optic tunable filter to select the operating wavelength. For the imaging system, a ×50 extra-long working distance microscope objective lens with a numerical aperture of 0.65 and a tube lens with a focal distance of 20 cm were used to image planes of interest on a camera. To image the focusing pattern, we moved the sample around the focal point using a translation stage.

**Fig. 3:**
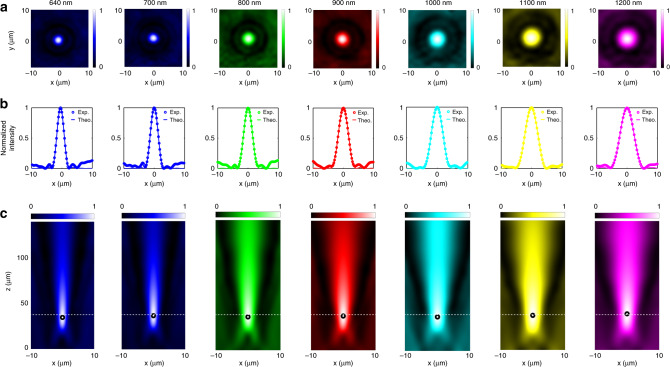
Experimental demonstration of achromatic and broadband focusing by a FAM. **a** Measured intensity profiles in the focal plane *z* = *F* (transverse *x*–*y* plane) of the FAM, with a numerical aperture NA = 0.12, at different wavelengths from 640 to 1200 nm. **b** Normalized cross-section of the experimental measurements in  **a** and the lines correspond to the theoretical airy disk. **c** Normalized intensity profiles in the plane *y* = 0 (axial *x–z* plane) around the focal point of the FAM at different wavelengths. Black circles represent the focal spots for different wavelengths from 640 to 1200 nm.

**Fig. 4:**
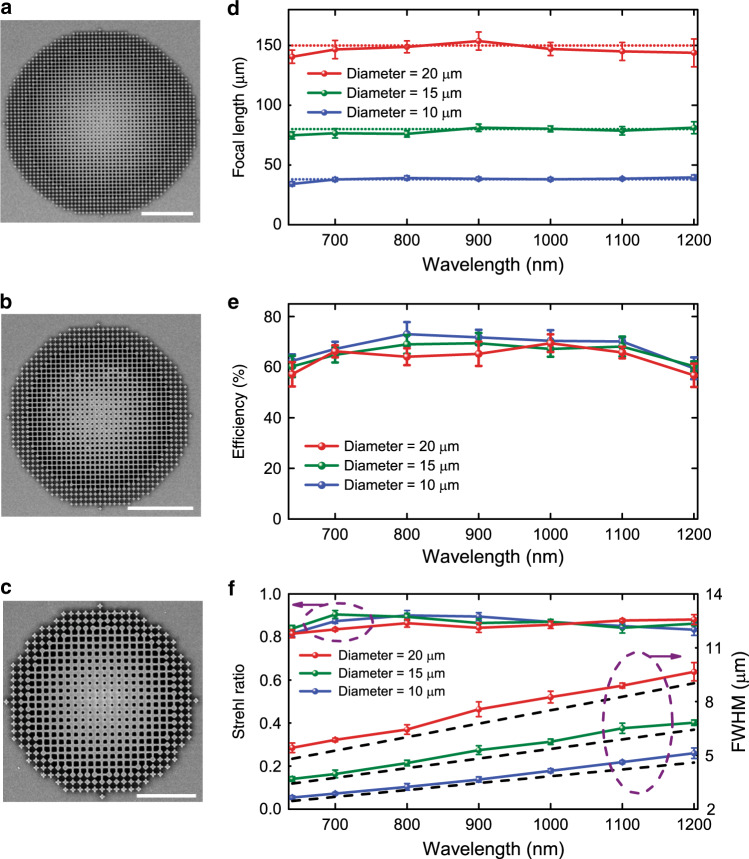
Measured performance of various FAMs. SEM of metalenses of diameter 20 µm **a**, 15 µm **b**, and 10 µm **c**. The scale bars in **a**–**c** represent 5, 5, and 3 µm, respectively. **d** Comparison of the measured and designed focal lengths for metalenses of various sizes. **e** Focusing efficiency and the measured average efficiency is about 70% in the band from 640 to 1200 nm. The measured efficiency of a FAM is defined as the power at the focal spot divided by the power transmitted through an aperture of the same diameter as the FAM. **f** FWHM at corresponding focal planes as a function of the wavelength (right axis) and measured Strehl ratios (left axis) of the FAMs. Results indicate that FAMs enable diffraction-limited focusing. Error bars are the standard deviation of measurements on different samples.

Figure 3a presents the measured intensity profiles in the focal plane *z* = *F* (transverse *x–y* plane) of the metalenses at different wavelengths. The dots in Fig. 3b represent a normalized cross-section of the experimental measurements in Fig. 3b and the lines correspond to the theoretical Airy disk. Figure 3c shows the normalized intensity profiles in the plane *y* = 0 (axial *x*–*z* plane) around the focal point of the metalens at different wavelengths. Black circles represent the focal spots for different wavelengths. These results show nearly diffraction-limited focal spots with no obvious distortion. To further examine the performance of designed metalenses, we measured focal lengths, focusing efficiencies, and full widths at half maximum (FWHM) for different lens diameters as shown in Fig. 4. Figure 4a–c present SEM images of lenses of diameters 10, 15, and 20 μm. Figure 4d presents the focal length of the metalenses and shows that they are mostly unchanged when the wavelength varies from 640 to 1200 nm, demonstrating the successful realization of the broadband achromatic property. Figure 4e presents the focusing efficiency for metasurfaces of different diameters and focal lengths. To enable a quantitative comparison between our devices and previously reported metalenses, we defined the size of the focal spot as three times the FWHM in the measurements of the efficiencies^9,16,36^. The measured efficiency of a metalens is defined as the focal spot power divided by the transmitted power through an aperture of the same diameter as the metalens. Efficiencies from 65% to 75% for the entire band are measured. In Fig. 4f (right axis), the experimental FWHM plots of the focal spots are presented. We also present in Fig. 4f (left axis) the Strehl ratio defined as the ratio of the peak focal spot irradiance of the manufactured FAMs to the focal spot irradiance of an aberration-free lens. The calculation includes the total energy enclosed by the measured focal spot within a diameter up to the second dark ring of corresponding airy disk. These results show that FAMs successfully achieve a diffraction-limited focus. Similar results are obtained for the *X* and *Y* polarization confirming polarization independence (Supplementary Fig. 8). Beyond the extremities of the current bandwidth, the slopes are smaller than the target slopes at different positions owing to the dispersion of TiO_2_ and leading to an increased focal length. We have thus successfully implemented planar achromatic metalenses spanning the continuous wavelength range from 640 nm in the visible to 1200 nm in the infrared.

## Discussion

To compare metasurfaces operating in various wavelengths range, a fair metric is the fractional bandwidth defined as the bandwidth divided by the central frequency, i.e., Δ*λ*/*λ*_center_ = Δ*f*/*f*_center_ with Δ*λ* = *λ*_max_ − *λ*_min_ and *λ*_center_ = (*λ*_max_ + *λ*_min_)/2. Our FAMs have a fractional bandwidth of 61% with an efficiency of 70%. The fractional bandwidth in ref. ^14^ is 35% with an efficiency smaller than 20%. In ref. ^15^, the fractional bandwidth is 41% with an efficiency smaller than 35%. To the best of our knowledge, the FAMs have higher efficiencies and larger fractional bandwidths than other experimentally reported metasurfaces. Moreover, compared to multi-level diffractive lenses presented in ref. ^36^, the FAMs can be extended to anisotropic structures to enable functions not easily achieved with diffractive elements. Metalenses have the advantage to enable subwavelength unit-cells which usually come at the price of the bandwidth and efficiency and this tradeoff is overcome in our design. Large scale metalenses are of technological importance. FAMs can be implemented at larger scale by increasing the maximum slope which will require higher aspect ratio. State of the art diffractive optics experiments have an efficiency of 35% and a fractional bandwidth of 62.7%^37^. It is worth noting that metalenses have larger angular transmission compared to Fresnel lenses, which suffer from the shadowing effect due to their sawtooth surface profile^38^. Our work thus brings metasurfaces to a performance level not previously reached.

We proposed and experimentally demonstrated metalenses combining high efficiency, polarization independence, and achromaticity in the continuous wavelength range from 640 nm in the visible to 1200 nm in the infrared. The broadband operation is achieved by enforcing the slopes of the phase-shift that vary continuously from the center of the lens to its edge, and, by minimizing the phase-shift intercepts that are ideally zero for achromatic operation. To the best of our knowledge, this is the broadest band achromatic metalens reported to date. The proposed approach significantly extends the current state of the art of metalenses both in terms of bandwidth and efficiency and opens the door to many applications.

## Methods

### Numerical simulations

Numerical simulations are performed using finit-element method (CST Microwave Studio) and the local phase method, followed by least-square linear fitting. The phase shift of elements is calculated using a reference at the center of the lens with geometric parameters *W* = 270 nm, *R* = 135 nm, and *L* = *P* = 370 nm.

### Sample fabrication

The fabrication flow chart is shown in Supplementary Fig. 3. An electron beam resist (EBR) [370 nm PMMA A4] is coated on the sample (1500 rpm) followed by 1 min bake at 180 °C (hot plate). The metasurface pattern is written in the resist using EBL. We patterned the resist using EBL followed by a development process to remove the exposed EBR. This resist pattern is the inverse of our final metasurface. We transferred the patterned sample to an ALD chamber at low temperature. The low-temperature deposition is important to obtain the desired amorphous material and to avoid the contamination of the ALD chamber. Using planarization, we removed the residual TiO_2_ film on top of the surface of the resist by reactive-ion-etching using a mixture of BCl_3_ and Cl_2_ gases. The etch depth was equal to *t*_film_ so that the etching process exposes the underlying resist and the top of the nanostructures. We finally removed the remaining EBR and left only the TiO_2_ metasurfaces.

### Optical measurements

To optically characterize the fabricated lenses, a custom setup was used. The experimental setup (Supplementary Fig. 5) is composed of two main systems dedicated to illumination and imaging. The illumination system comprises a supercontinuum laser (NKT photonics) and an acousto-optic tunable filter (Super K) to select the operating wavelength from 640 to 1200 nm illuminating 100% of the lens surface. For the imaging system, a ×50 extra-long working distance microscope objective lens with a NA = 0.65 and a tube lens with a focal distance of 20 cm were used to image intensity at planes of interest with a camera. To image different planes of the sample, we moved the sample using a translation stage around the focal point.

## Supplementary information

Supplementary Information

## Data Availability

The data that support the plots within this paper and other findings of this study are available from the corresponding author upon reasonable request.

## Code availability

The computer codes that support the plots within this paper and other findings of this study are available from the corresponding author upon reasonable request.
