# Supplementary material for: Octave bandwidth photonic fishnet-achromatic-metalens
Source: Nat Commun. 2020 Jun 25;11:3205. doi: 10.1038/s41467-020-17015-9 (PMC7316784; doi:10.1038/s41467-020-17015-9)
Supplement: Supplementary file 1 — Supplementary Information [file 41467_2020_17015_MOESM1_ESM.pdf]

Supplementary Information

Octave bandwidth photonic fishnet-achromatic-metalens

Abdoulaye Ndao[1,2*], Liyi Hsu [1,2*], Jeongho Ha[1,2], Jun-Hee Park [1,2], Connie Chang-Hasnain[1], and Boubacar Kanté [1,2,3,4]

[1] *Department of Electrical Engineering and Computer Sciences, University of California, Berkeley, California 94720, USA*

[2] *Department of Electrical and Computer Engineering, University of California San Diego, La Jolla, California 92093-0407, USA*

[3]*Materials Sciences Division, Lawrence Berkeley National Laboratory, 1 Cyclotron Road, Berkeley, California 94720, USA*

[4]*Department of Mechanical Engineering, University of California, Berkeley, California 94720, USA*

bkante@berkeley.edu

*These authors contributed equally to this work

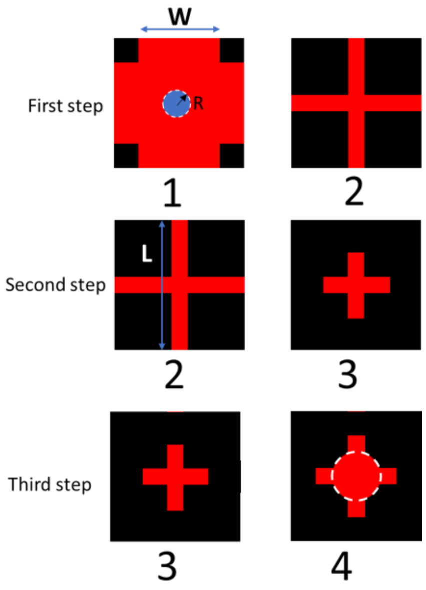

Supplementary Figure 1: Flow chart of the design and evolution of the unit-cell. In the first step, we changed the radius R and the width (W). When the width is larger than the diameter (W>2R), the change of the radius cannot be seen in the geometry as the cylinder is embedded in the square (see first step of the flow chart of the design). Therefore, the region W>2R is shadowed as only W is relevant in that region. In the second step, we first changed the length of the bridge and for the third step added a cylinder which gives a new degree of freedom and we changed the radius (third steps of the flow chart).

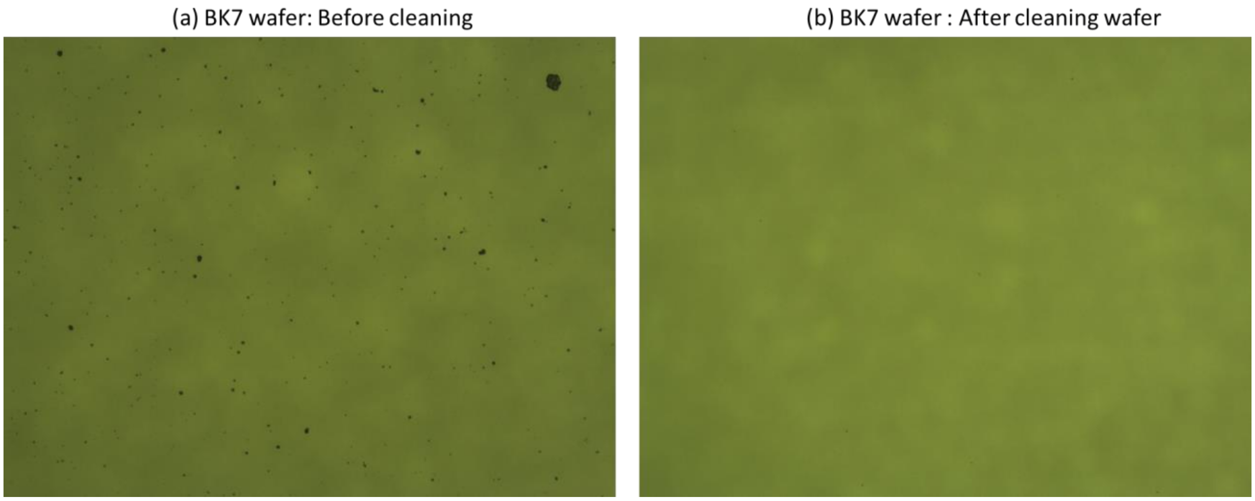

Supplementary Figure 2. Cleaning method. BK7 wafer before cleaning (a), and after cleaning (b). First, it is crucial to thoroughly clean the BK7 glass substrate in order to acquire a surface as clean as possible for the subsequent deposition steps. The glass substrate is immersed in an acetone bath to remove any organic traces and then transferred to an isopropyl alcohol-containing beaker to remove the remaining dirt of any origin . An optical microscope image of the samples before cleaning 2(a) and after cleaning(b).

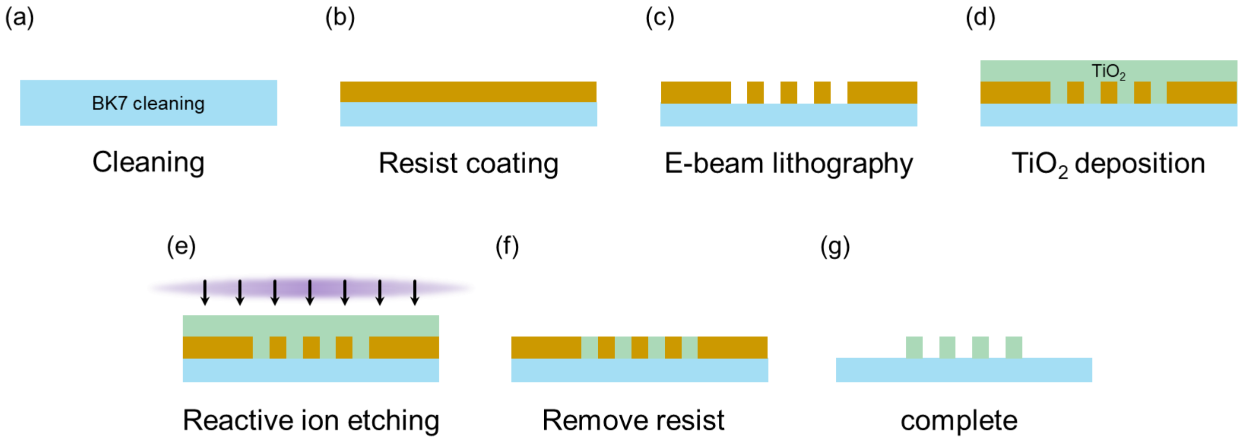

Supplementary Figure 3. Fabrication flow chart of the FAMs. The flow chart of a metasurface fabrication using the top-down etching and lift-off process. In addition, O_2 plasma treatment performed to the glass substrate for adhesion between glass and other materials including resist and TiO_2. An electron beam resist (370nm PMMA A4) is coated on the sample (1500 rpm) followed by 1min bake at 180 °C (hot plate). The metasurface pattern is written in the resist using electron beam lithography (EBL) (step c)). We patterned the resist using electron beam lithography then development process performed to remove the exposed EBR. This resist pattern is the inverse of our final metasurface. We transferred the patterned sample to an atomic layer deposition (ALD) chamber. The low temperature deposition is important to obtain the desired

amorphous material and also to avoid the contamination of the ALD chamber by the EBR. Using planarization method, we removed the residual TiO_2 film on top of the surface of the resist by reactive-ion-etching using applying mixture of BCl_3 and Cl_2 gases. The etch depth was equal to t_{film} so that the etching process exposes the underlying resist and the top of the nanostructures. We finally removed the remaining EBR (step g) and left only the TiO_2 metasurfaces.

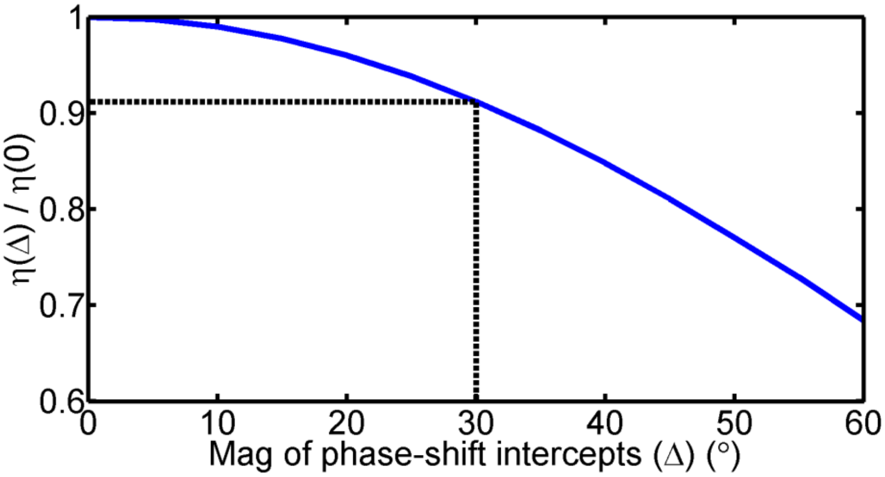

Supplementary Figure 4. Efficiency change with non-zero phase-shift intercepts in the metalens Monte Carlo simulations are performed with hundred simulations for each element in the metalens by using a homemade finite difference time domain code. Each simulation was given a certain magnitude of the phase-shift intercept (error or deviation from the ideally zero phase-shift intercept) that was randomly distributed between unit-cells. The focusing efficiency ($\eta(\Delta)$) is then compared to the ideal metalens ($\eta(0)$) implementing not only the correct slope but also the correct phase-shift intercept. Efficiency ratio between the metasurface with randomly distributed non-zero phase-shift intercept ($\eta\ (\Delta)$) and the one with zero phase-shift intercept ($\eta\ (0)$).

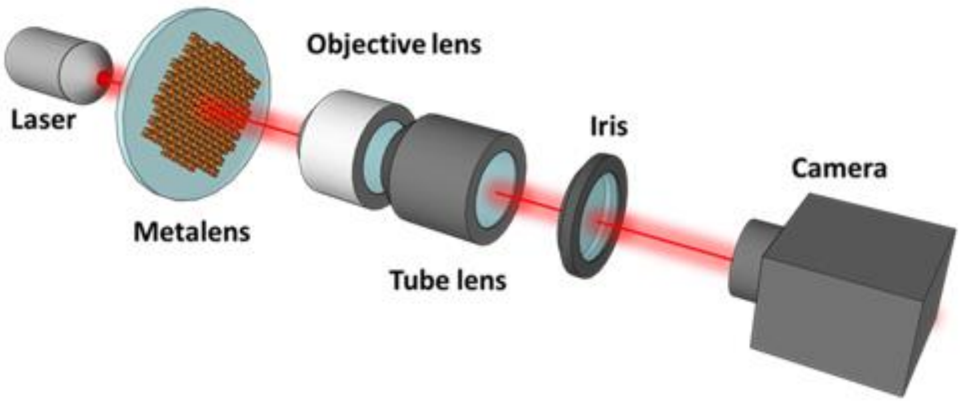

Supplementary Figure 5. Experimental setup. The experimental setup is composed of two main systems dedicated to illumination and imaging. The illumination system comprises a supercontinuum laser (NKT photonics) and an acousto-optic tunable filter (Super K) to select the operating wavelength from 640 nm to 1200 nm with bandwidth around 10 nm illuminating 100% of the lens surface. For the imaging system, a 50X extra-long working distance microscope objective lens with a NA =0.65 and a tube lens with a focal distance of 20 cm were used to image intensity at planes of interest to a camera. To image different planes of the sample, we moved the sample using translation stage around the focal point.

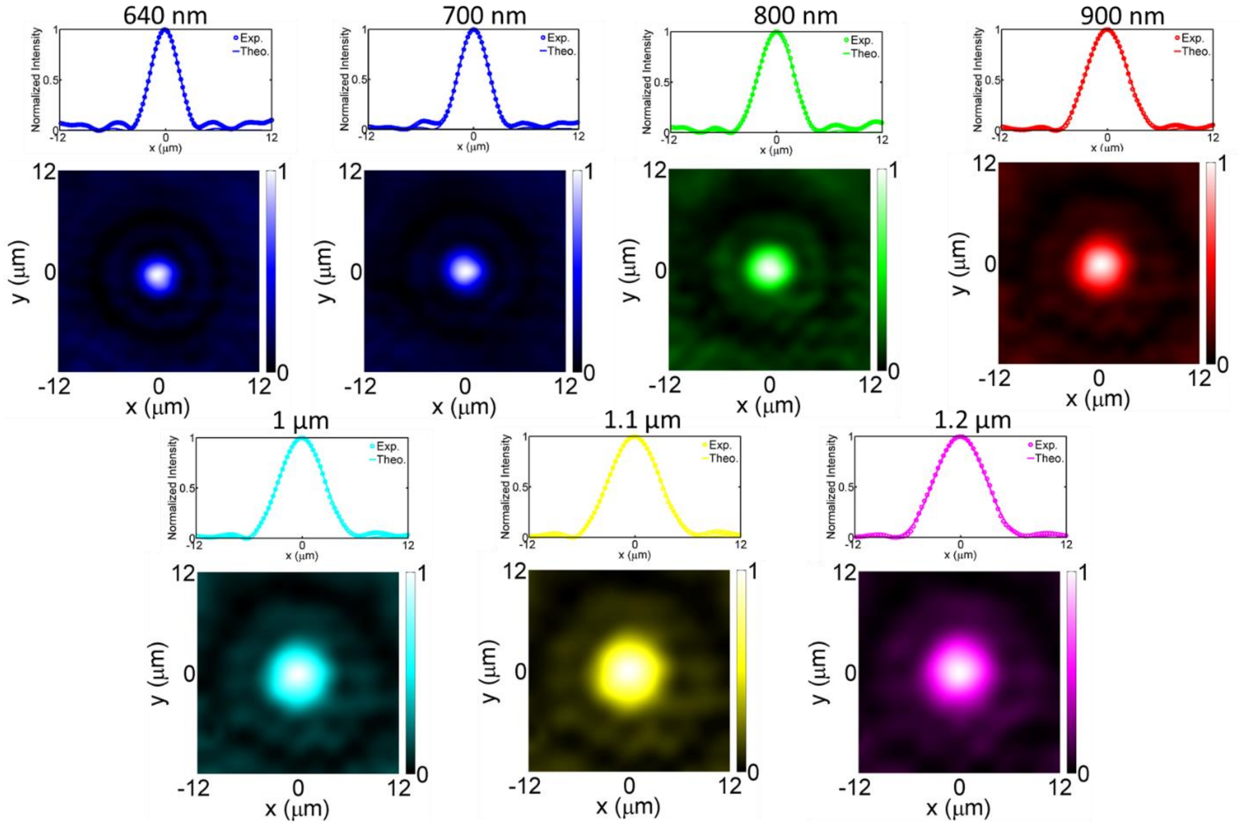

Supplementary Figure 6.Normalized intensity profiles at the focal point. Measured intensity profiles (1D and 2D) of the achromatic metalens at different wavelengths from 640 nm to 1200 nm (Diameter = 15 μm) with NA=0.1. The circles are the measurement and the lines are from an ideal Airy spot.

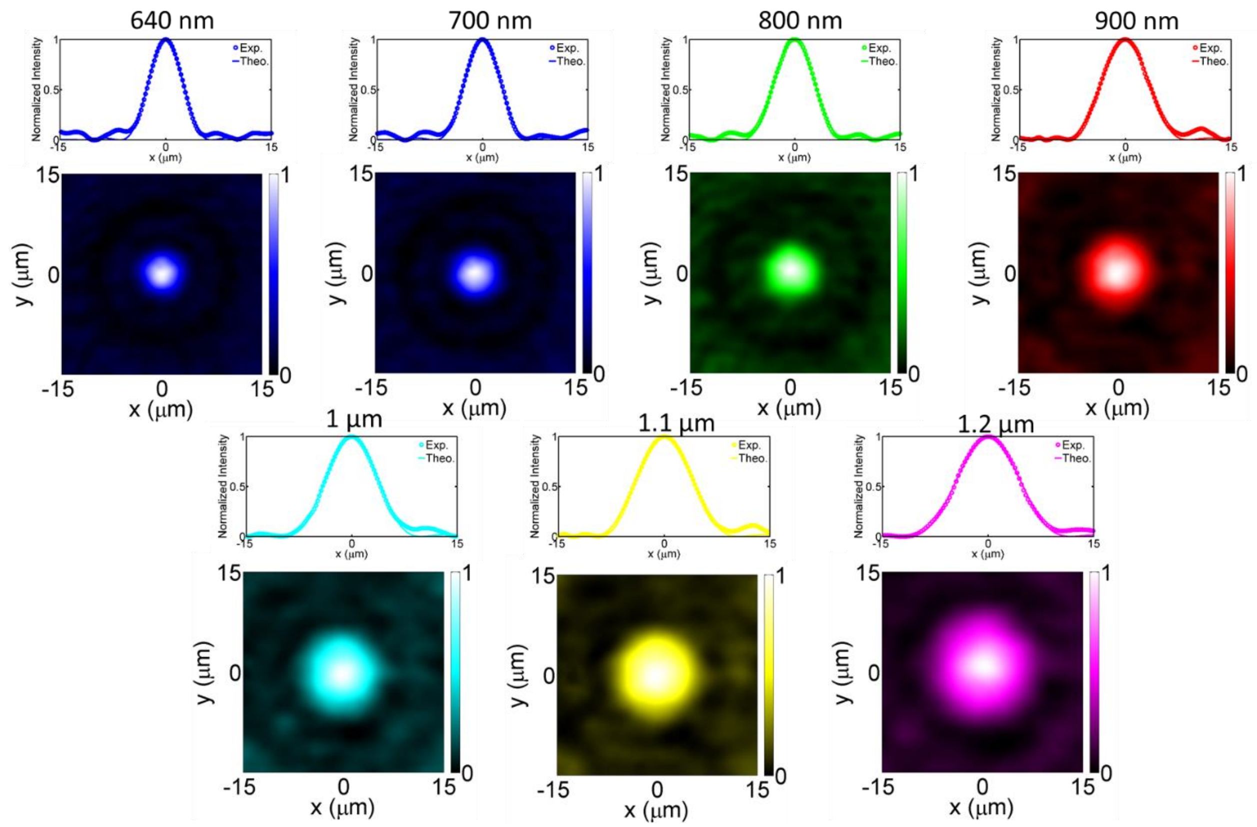

Supplementary Figure 7: Normalized intensity profiles at the focal point. . Measured intensity profiles (1D and 2D) of the achromatic metalens at different wavelengths from 640 nm to 1200 nm (Diameter = 20 μm) with NA=0.077. The circles are the measurement and the lines are from an ideal Airy spot.

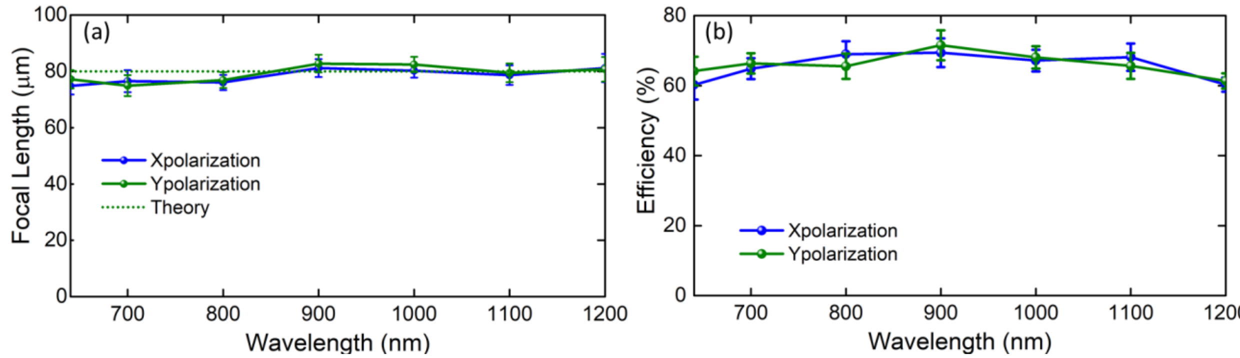

Supplementary Figure 8: Performance of the FAM for different polarizations. (a) Measured focal length of a FAM for the X and Y polarization. (b) Efficiency from 640 nm to 1200 nm for the two polarizations. Error bars are the standard deviation of measurements on different samples.

Results show that the focal length (a) and the efficiency (b) of the metalens are unchanged when polarization is varied.

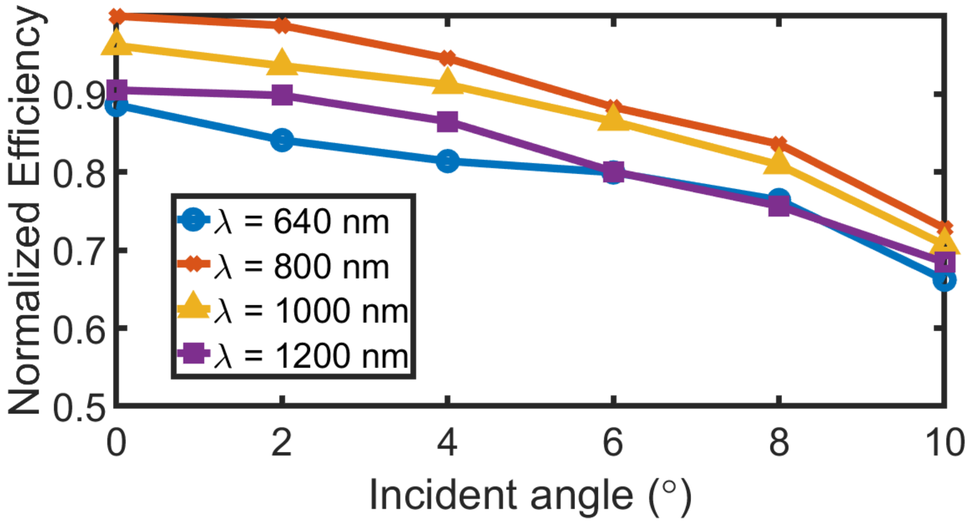

Supplementary Figure 9. Normalized efficiency as a function the incident angle for various wavelengths. The normalized efficiency as a function of the incidence angle at various wavelengths. As seen, the efficiency decreases by about 30% for 10° incidence angle. This shows that the current design is sensitive to the incidence angle.

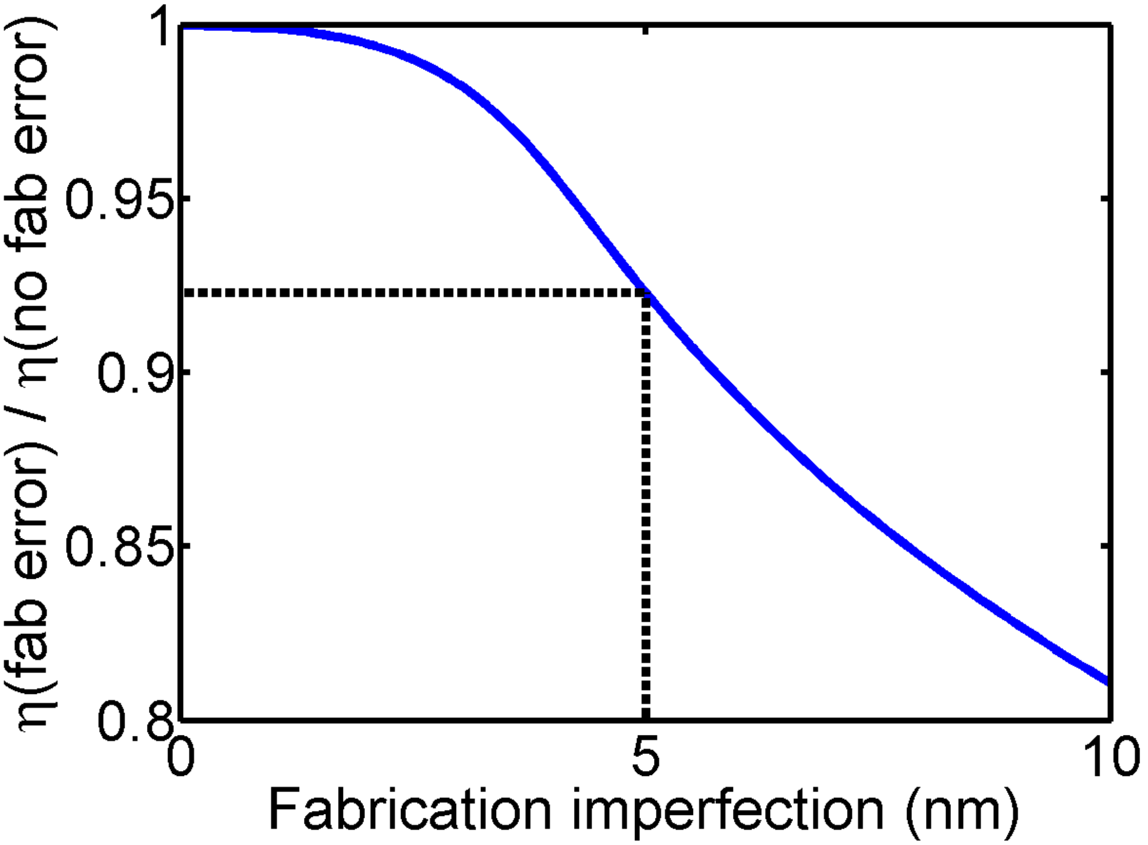

Supplementary Figure 10. Fabrication imperfections. Efficiency ratio between the metasurface with fabrication errors of amplitude ±5nm and the metasurface without fabrication errors. We investigated imperfections in fabrication as a function of various phase errors at different positions and considered fabrication imperfection originating from rounded corners. When dimensions of the FAM are varied by ±5nm compared to the designed dimensions, the phase of unit-cells from the center to the edge of the metalens changes by less than 28 deg. We further evaluated the effect of such fabrication errors on the performance of the FAM using Monte Carlo simulations using a homemade finite difference time domain code. Each simulation was given a certain magnitude of the phase-shift (error or deviation from the with the correct dimensions (η(no fab error)) is about 28 deg that was randomly distributed between unit-cells. The focusing efficiency (η(Δ)) is then compared to the metalens with the correct dimensions (η(no fab error)) implementing the correct dimension. The variation of the geometrical parameters by ±5nm shows that efficiency decrease by at most 8%. The design is thus robust.

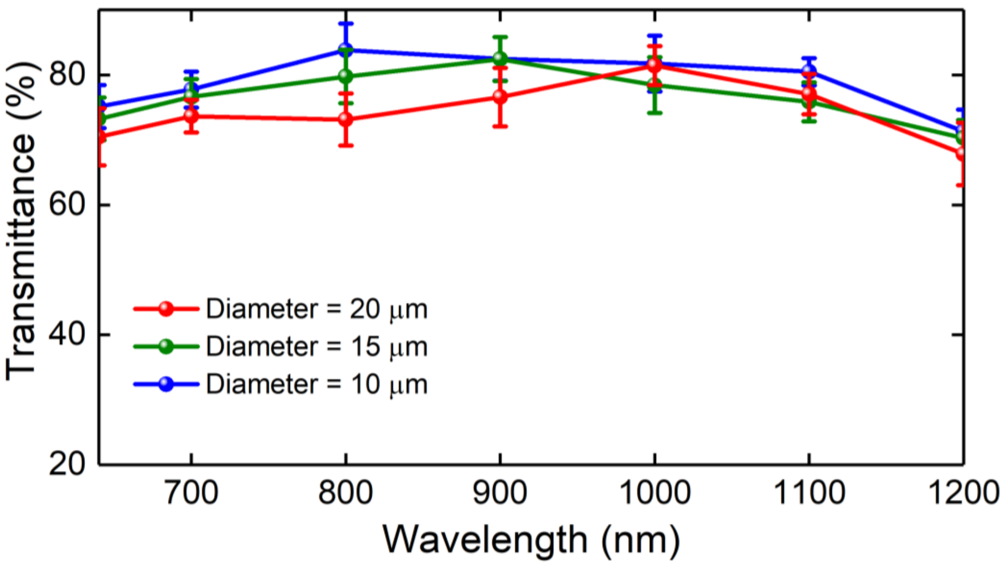

Supplementary Figure 11. Transmittance of the three fabricated FAMs. Error bars are the standard deviation of measurements on different samples.
